# Tailoring the Intersystem Crossing and Triplet Dynamics of Free-Base Octaalkyl-β-oxo-Substituted Porphyrins: Competing Effects of Spin–Vibronic and NH Tautomerism Relaxation Channels

**DOI:** 10.1021/acs.jpca.2c01225

**Published:** 2022-03-29

**Authors:** Sayantan Bhattacharya, Arthur Graf, Anna Karolyna M. S. Gomes, Nivedita Chaudhri, Dimitri Chekulaev, Christian Brückner, Thiago M. Cardozo, Adrien A. P. Chauvet

**Affiliations:** † Department of Chemistry, The University of Sheffield, Sheffield S3 7HF, United Kingdom; ‡ Instituto de Química (IQ), Federal University of Rio de Janeiro, Rio de Janeiro 21941-909, Brazil; § Department of Chemistry, University of Connecticut, Storrs, Connecticut 06269-3060, United States

## Abstract

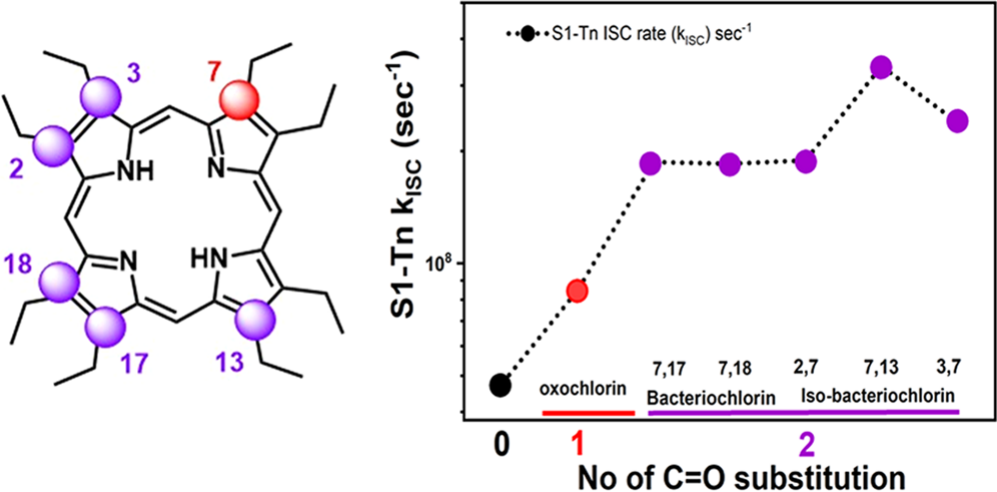

We demonstrate that β-oxo-substitution provides effective fine-tuning of both steady-state and transient electronic properties of octaalkyl-β-mono-oxochlorin and all isomers of the β,β′-dioxo-substituted chromophores. The addition of a carbonyl group increases the Q*_y_* oscillator strength and red-shifts the absorption spectra. Each oxo-substitution results in a 2-fold increase in the singlet to triplet state intersystem crossing (ISC) rates, resulting in a 4-fold ISC rate increase for the dioxo-substituted chromophores. The effects of oxo-substitution on the ISC rate are thus additive. The progressive increase in the ISC rates correlates directly with the spin–vibronic channels provided by the C=O out-of-plane distortion modes, as evidenced by density functional theory (DFT) modeling. The triplet states, however, were not evenly affected by β-oxo-substitution, and reduction in the triplet lifetime seems to be influenced instead by the presence of NH tautomers in the dioxoisobacteriochlorins.

## 1 Introduction

Porphyrins play a central role in catalyzing a wide variety of crucial reactions in living organisms, including photosynthesis and CO_2_ fixation.^1−4^ As these reactions address some of the world’s most pressing environmental challenges, huge efforts in mimicking these processes have been undertaken in the past decades. Unsurprisingly, porphyrins also play a key role in these synthetic systems.^5,6^ Because of their high absorption cross section and versatile synthesis, porphyrins are ideal light harvesters whose electronic properties can be conveniently tuned.^7,8^

This work focuses on the processes and modifications that enhance intersystem crossing (ISC) rates as well as modulate triplet states, which are sought in most applications such as sensitizers, photodynamic therapeutic materials, etc.^6−8^ The substitution of heavy atoms such as Br, I, and S has been found to be highly effective in enhancing ISC rates.^9^ Such modifications, however, increase the toxicity of porphyrins and make them unsuitable for most biologically relevant applications.

In addition to using heavy and toxic metals and bulky substituents, the incorporation of more convenient functional groups such as carbonyl, nitro, or diazine has also been found to impact ISC rates in organic molecules. More specifically, this tuning is achieved through the effective modification of spin–orbit coupling between the low-lying n−π* and π–π* states of the molecules.^9,10^ Among the various functional groups available, the introduction of the auxochrome β-oxo-functionalities to porphyrinoids was already shown to be an appropriate alternative.^11−17^ And although the tunability of transient properties via the incorporation of the carbonyl group is not guaranteed,^15^ it has been shown to be effective in a wide variety of compounds and materials.^18−20^ Accordingly, the introduction of β-oxo-functionalities is expected to act through the spin–vibronic mechanism, which has been shown to be detrimental for intersystem crossing (ISC) in molecules.^12,21^

**Figure 1:**
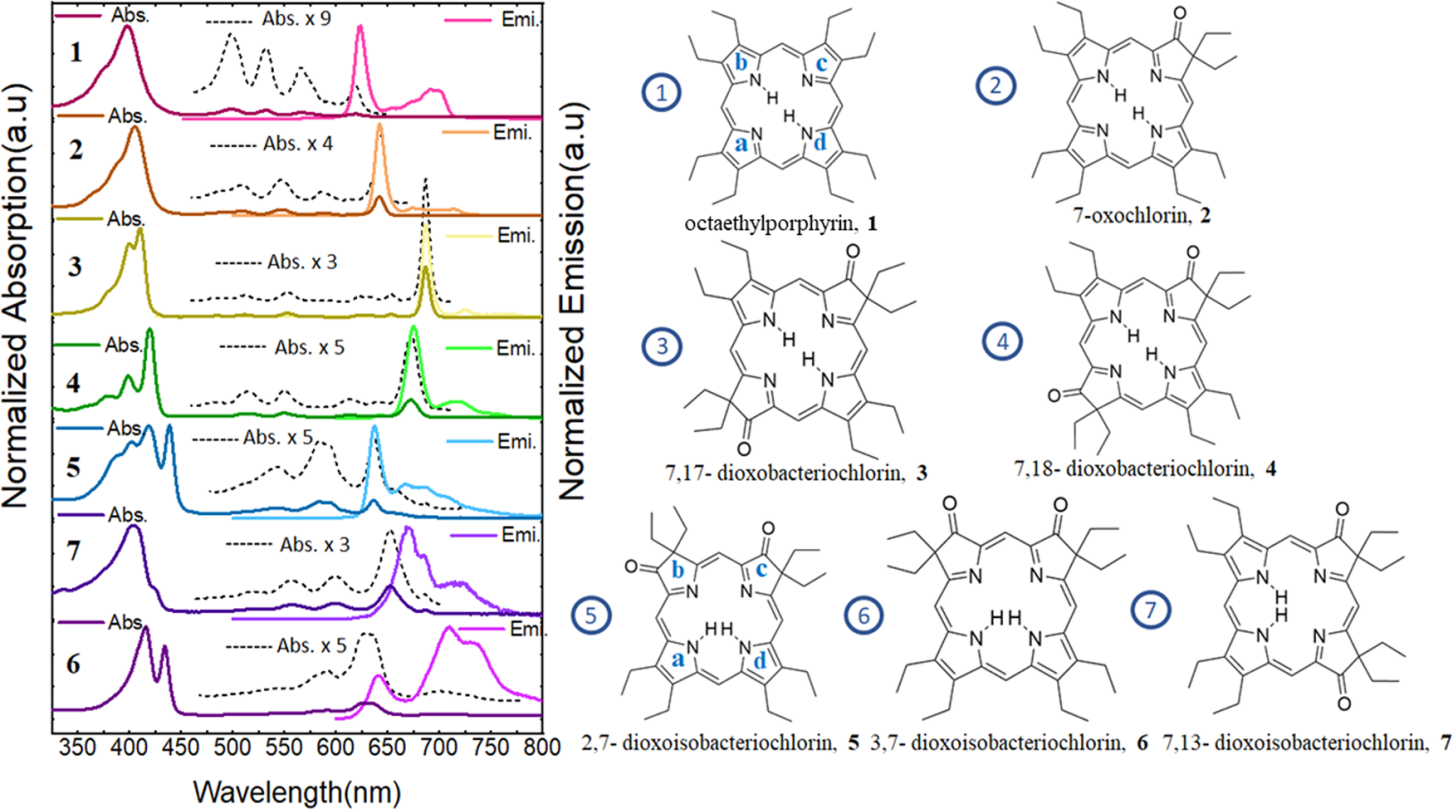
Molecular structures and the corresponding normalized absorption and emission spectra for all oxochlorin, dioxobacteriochlorins, and isobacteriochlorins. Structures of the most stable ground-state NH tautomers for the dioxoisobacteriochlorin isomers **5**–**7**, as shown in our previous work.^25^ Note that the naming “chlorin,” “bacteriochlorin,” etc. describes the relative position of the oxo-substituents and is not a reflection of the electronic structure of the chromophores.

The simplest synthetic methodology to introduce β-oxo-functionalities into synthetic β-alkylporphyrins is to treat them with H_2_O_2_ in concentrated H_2_SO_4_.^22−24^ Using octaethylporphyrin (OEP, **1**), this reaction allows the preparation of oxochlorin **2** and all regioisomers of the dioxo-derivatives shown in Figure 1: the two isomers of the dioxobacteriochlorin series **3** and **4** and the three possible isomers of the dioxoisobacteriochlorin series **5** through **7**.^25^

In this study, the properties of all octaethyl-β-oxo-substituted porphyrinoids were compared to the benchmark compound OEP (**1**) and were investigated by means of steady-state absorption and emission spectroscopies. The electron dynamics of porphyrins were investigated by means of time-correlated single-photon counting (TCSPC) and ultrafast transient absorption spectroscopy in the range from femto- up to milliseconds. We demonstrate that β-oxo-substitution provides effective fine-tuning of both the steady-state and transient electronic properties of octaalkyl-β-mono-oxo and dioxo-porphyrins. In terms of steady-state properties, each addition of a carbonyl group increases the Q*_y_* oscillator strength and red-shifts the whole absorption spectra (Soret and Q-bands). In terms of transient properties, each oxo-substitution results in a 2-fold increase in the singlet to triplet state ISC rates, resulting in a 4-fold ISC rate increase for the doubly β-oxo-substituted chromophores when compared to OEP. The progressive increase in the ISC rates correlates directly with the increased spin–vibronic channels provided by the C=O out-of-plane distortion modes, as evidenced by density functional theory (DFT) modeling. The triplet lifetime, however, does not seem to be uniformly affected by the presence of carbonyl groups but is interestingly linked to the presence of stable ground-state NH tautomers.

We are thus witnessing a direct competition between the C=O out-of-plane distortion spin–vibronic channels and the presence of ground-state NH tautomers in determining the excited-state dynamics of the β-oxo-chromophores investigated. As a result, access to such a unique series of porphyrins also gives us the opportunity to show how NH tautomerism competes with the spin–vibronic channel in tuning the porphyrinoid photophysics. This study thus defines the scopes and limits of β-oxo substitution in tuning the excited-state dynamics. Accordingly, this study provides insights into designing porphyrinoids for specific applications.

## 2 Experimental Section

### 2.1 Materials

We have chosen free-base octaethylporphyrin OEP **1**, oxochlorin **2**, dioxobacteriochlorin isomers **3** and **4**, and dioxoisobacteriochlorin isomers **5**–**7** for our measurements (as shown in Figure 1). Syntheses and structural characterizations of the β-oxo-chromophores **2**–**7** of the compounds **2**–**7** have been reported elsewhere.^25^ All photophysical measurements were performed in dichloromethane (CH_2_Cl_2_).

### 2.2 Photophysical Methods

Steady-state UV–vis and fluorescence measurements were performed using a Cary 60 UV–vis spectrometer and FluroMax spectrophotometer, respectively. Fluorescence quantum yields were measured using a FluroMax spectrometer. Time-resolved fluorescence data were collected on a TCSPC system of Edinburgh instrument FS5 spectrofluorometer; samples were kept in a 1 cm transparent quartz cuvette, and a NanoLED of 365 nm was used as an excitation source, with an instrument response function of ∼500 ps.

To avoid the generation of singlet oxygen by quenching of triplet states, all porphyrin samples were degassed using the freeze–pump–thaw method before transient measurements study. The samples were held in 2 mm path length degassing quartz cells and were continuously stirred during experiments using a magnetic stirring system. The optical density at the excitation wavelength was kept at ∼0.3. Transient absorption (TA) spectroscopy was performed at the Lord Porter Laser Laboratory, University of Sheffield, in fs–ps and ns–ms regimes separately. fs–ps TA was performed using a commercial transient absorption spectrometer (Helios, Ultrafast Systems) using a CMOS sensor for the UV–vis spectral range, where a Ti:Sapphire regenerative amplifier (Spitfire ACE PA-40, Spectra Physics) was used as the main laser source. Excitation under a pump wavelength of 400 nm was generated using the second-harmonic generation of the 800 nm output in a β-BBO crystal within a commercially available higher harmonic generator (TimePlate, Photop Technologies). White light supercontinuum (WL) probe pulses were generated using a CaF2 crystal (in the range of 340–790 nm). The relative polarization of the pump and probe pulses was set to the magic angle of 54.7° for the measurements. Residual 800 nm in the WL is avoided by putting an 800 nm, 0° AOI hot mirror before focusing the WL onto the sample.

Ns–ms TA was performed with a home-built setup. All measurements were carried in a degassed solution with a constant magnetic stirring mechanism. A WL probe was generated by focusing 800 nm, 130 fs pulses at 1 kHz from Solstice (Ti:Sapphire regenerative amplifier) to a sapphire crystal, whereas the pump beam was achieved using a third-harmonic of an externally triggered InnoLas piccolo (diode pump Nd:YAG laser) laser operating at 500 Hz emitting 355 nm. The delay between the pump and probe was modulated using an electronic delay generator (Stanford research). Probe referencing was used to improve the signal-to-noise ratio. Probe and probe reference beams were dispersed by a volume phase holographic grating (Wasatch Photonics) and were detected by two linear Si-CCD arrays. The maximum achievable delay using this setup is ∼1 ms with a temporal resolution of ∼1 ns. Intersystem crossing yield (ϕ_ISC_) was calculated from ns–ms transient absorption kinetics. To measure the yield of S_1_ → T_n_ intersystem crossing, we compared the extent of bleaching of the ground-state absorption bands due to T_n_ at the asymptote of the S_1_ decay with the extent due to S_1_ immediately after the excitation.

### 2.3 Theoretical Methods

Both ground-state and excited-state geometry optimizations were performed at the B3LYP/def2-SV(P) level. Excitation energies were obtained at the time-dependent DFT (TDDFT) level of theory using the B3LYP and the def2-SVPD basis sets. Relaxed scans were used to obtain potential energy curves for the stretching movement of the C–C bond connected to the carbonyl group in oxochlorin **2** and the corresponding C–C bond in OEP. Relaxed scans were carried out in the same manner for the out-of-plane carbonyl displacement. Both DFT and TDDFT calculations were performed using TURBOMOLE 6.6 software.^26^ Spin–orbit coupling matrix elements (SOCMEs) between singlets and triplets were evaluated at the B3LYP/def2-SV(P) level using ORCA 4.2.12.^27^ Furthermore, the state-averaged CAS (4,4) and CAS (8,6) calculations using def2-SV(P) and def2-SVPD basis sets were performed to validate our TDDFT results using ORCA 4.2.1.

## 3 Results and Discussion

### 3.1 Absorption and Emission Spectra

**Figure 2:**
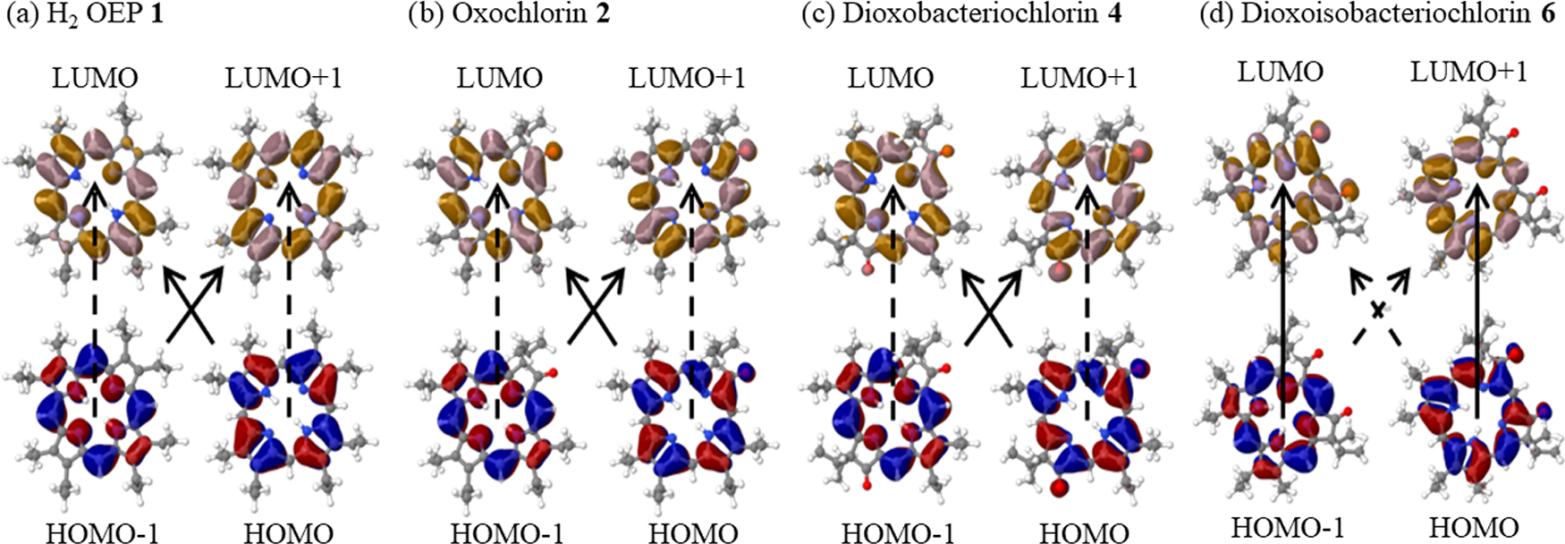
Orbitals associated with Q-band transitions obtained at the B3LYP/def2-SVPD level for (a) H_2_ OEP **1**, (b) oxochlorin **2**, (c) dioxobacteriochlorin **4**, and (d) dioxoisobacteriochlorin **6**. Full arrows represent S_0_ → S_1_ transitions and dashed arrows correspond to S_0_ → S_2_ transitions.

Both steady-state absorption and emission as well as time-resolved optical spectroscopy have been used to characterize molecules **1–7**. In terms of steady-state properties, the absorption spectra of molecules **1**–**5** and **7** follow Gouterman’s four-orbital model:^28,29^ An intense B (Soret) band in the 400–440 nm region is accompanied by weaker Q-bands in the 480–730 nm region (Figure 1).^28,30,31^ The stark differences in the nature of the spectra of the dioxoisobacteriochlorins (**5**–**7**) and dioxobacteriochlorins (**3** and **4**) highlight that the regiochemistry of the β-oxo substituents plays a significant role.^25^ DFT calculations were performed on OEP **1** and oxochlorin **2** to find out the shape of frontier molecular orbitals. Similar calculations were performed on bacteriochlorin **4** and dioxoisobacteriochlorin **6** as representative of the bacteriochlorin and isobacteriochlorin isomers. TDDFT results of the vertical excitations and oscillator strengths for OEP **1**, oxochlorin **2**, dioxobacteriochlorin **4**, and dioxoisobacteriochlorin **6** are presented in the Supporting Information Tables S-1–S-3. The calculated position for the lower energy Q*_y_*(0, 0) deviates by ∼60 to 80 nm when compared to the experimental values (see Table S-1), which is typical in that spectral range when using this method. The exception is dioxoisobacteriochlorin **6**, which shows a difference of ∼130 nm. The differences between calculated and experimental B-band positions are much smaller, ranging from ∼26 to 44 nm. The Q states are also predicted to be near-degenerate, which is in accordance with the observed superposition of the Q-band peaks. Orbitals associated with the Q-band transitions are similar to those proposed by Gouterman,^28^ except for, again, in the case of dioxoisobacteriochlorin **6**, where the orbitals present significant distortions toward the carbonyl groups, and there is an inversion in the nature of the Q-band states, as shown in Figure 2. Accordingly, transitions for molecule **6** cannot be fully described using the Gouterman four-frontier-orbital model as the two B-bands present non-negligible contributions from additional orbital transitions. This confirms the need for an extended orbital space to properly describe similar dioxoisobacteriochlorin chromophore systems.^32^

The addition of oxo-groups results in overall red shifts of the spectra compared to the benchmark porphyrin **1** and increases the Q*_y_*(0, 0) extinction coefficient (i.e., the absorption band at the highest wavelength) for all derivatives investigated. Increased Q*_y_*(0, 0) oscillator strength is consistent with the higher emission monitored for these porphyrins.^11^ Note that the Q*_x_*- and Q*_y_*-bands are clearly distinguishable for OEP **1**, oxochlorin **2**, and both isomers of dioxobacteriochlorins **3** and **4**. However, the Q-bands of dioxoisobacteriochlorins **5** through **7** appear to be broadened. The apparent spectral congestion in the Q-band region in dioxoisobacteriochlorins **5**–**7** is attributed to the presence of ground-state NH tautomers, each having its own unique absorption spectrum.^25^

Excitation in the Soret band of **1** and all other oxidized derivatives of OEP results in the emission in the red to near-IR regime, from 620 to 800 nm (Figure 1). Emission spectra show an intense sharp feature, followed by less-intense secondary peaks for OEP **1**, oxochlorin **2**, and dioxobacteriochlorins **3** and **4** with a negligible stokes shift with respect to the absorption spectra. On the contrary, dioxoisobacteriochlorin **6** exhibits prominent emission bands at 641 and 710 nm with increasing emission intensity. The intense Q*_y_*(0, 0) emission band centered at 622 nm for **1** peak gets red-shifted to 642, 686, and 675 nm for oxochlorin **2**, dioxobacteriochlorins **3** and **4**, respectively, in accordance with the absorption spectra. When excited at the Soret band, most chromophores emit with small Stokes shifts (∼1 nm), which hints at a minimally distorted excited state. Only dioxoisobacteriochlorins **6** and **7** show slightly larger Stokes shifts of 10–11 nm. The broader emission of the isobacteriochlorin isomers is also attributed to the presence of NH tautomers (see Figure S-2). Fluorescence quantum yields (ϕ_f_, in CH_2_Cl_2_) have been calculated for molecules **1**–**7** relative to that of relative to *meso*-tetraphenylporphyrin (TPP) (ϕ_f_ of TPP is 0.13 in CH_2_Cl_2_) (Table 1).^33^ Thus, the above discussion suggests that the steady-state optical properties of OEP can be effectively tailored by varying the number and the position of carbonyl groups as β-oxo substituents.

**Table 1:** Photophysical Parameters for OEP **1**, Oxochlorin **2**, Dioxobacteriochlorins **3** and **4**, and Dioxoisobacteriochlorins **5**–**7**

sample	τ_S_ (ns)	ϕ_f_^a^	ϕ_ISC_^b^	ϕ_IC_ = 1 – ϕ_f_ – ϕ_ISC_	∑*k*_nr_ (s^–1^)^c^	*k*_ISC_ (s^–1^)^d^
**1**	12.2 ± 0.06	0.14	0.58	0.28	1.18 × 10^7^	4.75 × 10^7^
**2**	5.42 ± 0.03	0.16	0.46	0.38	2.97 × 10^7^	8.48 × 10^7^
**3**	3.14 ± 0.06	0.15	0.59	0.26	4.65 × 10^7^	1.87 × 10^8^
**4**	2.75 ± 0.01	0.16	0.51	0.33	5.67 × 10^7^	1.85 × 10^8^
**5**	3.28 ± 0.08	0.12	0.62	0.25	3.81 × 10^7^	1.89 × 10^8^
**7**	2.46 ± 0.06	0.17	0.59	0.24	6.95 × 10^7^	2.39 × 10^8^
**6**	2.16 ± 0.03	0.14	0.73	0.13	6.53 × 10^7^	3.38 × 10^8^
	1.48 ± 0.03				9.52 × 10^7^	4.93 × 10^8^

a Fluorescence quantum yield was calculated relative to *meso*-tetraphenylporphyrin (TPP) in CH_2_Cl_2_ (ϕ_f_ of TPP was taken as 0.13 in CH_2_Cl_2_).^33^

b ϕ_ISC_ was calculated from ns–ms transient absorption kinetics. ϕ_ISC_ was calculated by comparing the ground-state bleach amplitude immediately after the excitation and its remaining amplitude in the 100 ns range.

c ∑*k*_nr_ = (1 – ϕ_f_)/τ_S_.

d *k*_ISC_= ϕ_ISC/_τ_S_.

### 3.2 Excited-State Dynamics

Spectrally resolved TCSPC has been used to determine the emission lifetime of porphyrin **1**, which decays monoexponentially with a lifetime of 11.7 ± 0.02 ns (see Figure S-2) and is in agreement with the previously reported result.^34^ In comparison, oxochlorin **2** has an emission lifetime of about one-half of that of porphyrin **(1**5.3 ± 0.05 ns). All dioxochromophores have emission lifetimes that are about one-fourth of that of **1**, ranging from 1.5 to 3.3 ns, as shown in Table 1 and Figure S-1. Note that isobacteriochlorin **6** has a biexponential emission with both lifetimes being within the above range. We attribute this biexponential emission decay to the presence of NH tautomers. Based on a correlation of TCSPC emission and singlet state emission yield, we have computed the nonradiative rate for all porphyrins using ∑*k*_nr_ = (1 – ϕ_f_)/τ_S_, where ϕ_f_ is the experimentally monitored fluorescence quantum yield and τ_S_ is the singlet lifetime.^35^ The values for ϕ_f_, τ_S_, and ∑*k*_nr_ are tabulated in Table 1. Thus, we may conclude that it is possible to tune the emission lifetime as well as the nonradiative rate by varying the number and the position of carbonyl substituents in free-base OEP.

#### 3.2.1 Transient Absorption Spectroscopy from fs to ns

We further investigated the tuning abilities of oxo-substitution in the excited-state dynamics of the β-oxo-porphyrinoids via TA spectroscopy. Femtosecond TA spectra were collected for all degassed samples in a CH_2_Cl_2_ solvent by exciting at the Soret band with a pump wavelength of 400 nm. Global fitting of kinetics from different probe regions with multiexponential model convoluted with instrument response function suggests a multiphasic relaxation process from all of the samples. It has been reported previously that the decay time of the Soret band for free-base porphyrin falls on the order of ∼40 fs.^36^ Furthermore, a fluorescence upconversion study on free-base porphyrin suggested that Q*_x_* relaxation occurs within hundreds of femtoseconds.^37^ Thus, even if we initially populate the S_2_ state (by exciting at ∼400 nm), given our ∼150 fs temporal resolution and given the ultrafast nature of the S_2_ to S_1_ transition (<100 fs),^36−38^ it is safe to assume that the excited-state TA spectra from the oxo-porphyrinoids originate mostly from the electron dynamics of the S_1_ state and subsequent photophysical processes such as ISC and electron dynamics of triplet states.

**Figure 3:**
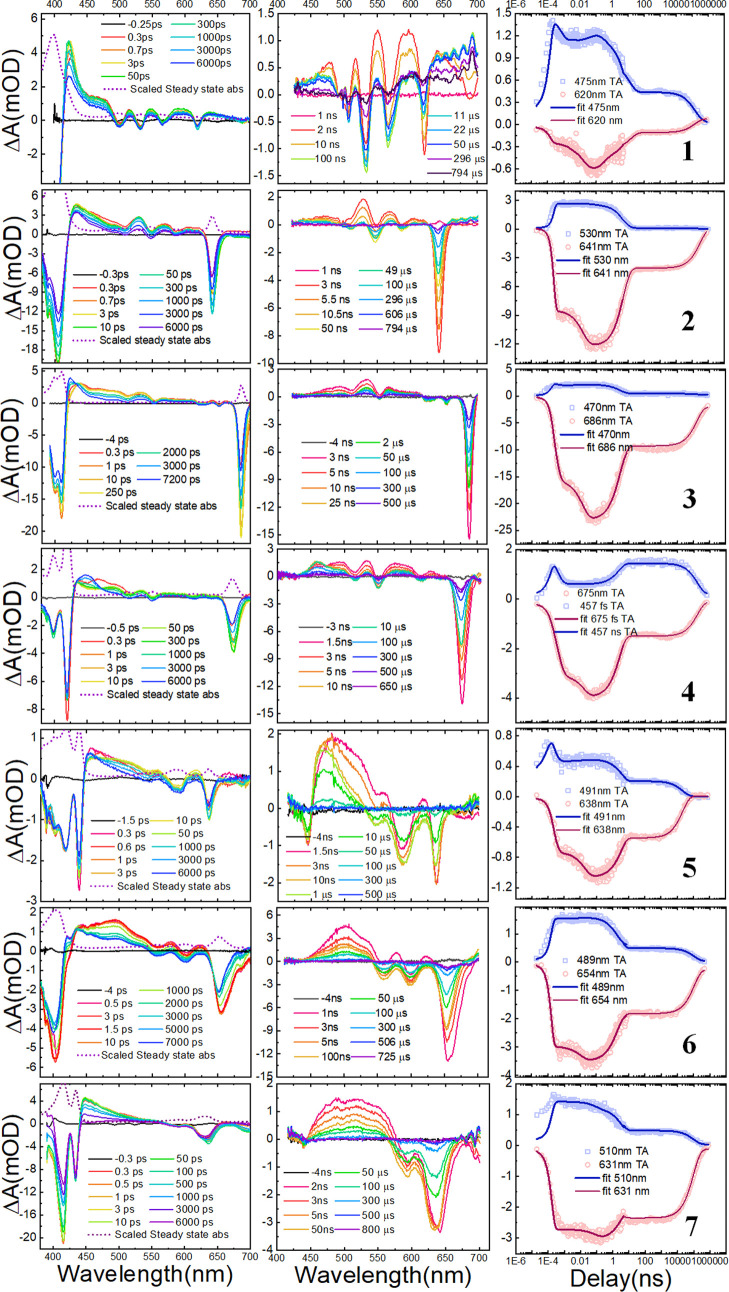
Transient absorption spectra in the fs–ns (left) and ns–ms regimes (middle) along with the full probe kinetics (right) of OEP **1**, dioxobacteriochlorins **3** and **4**, and dioxoisobacteriochlorins **5**–**7**.

TA spectra from the fs–ns regime and the ns–ms regime along with probe kinetics are shown in Figure 3. TA spectra of all of the porphyrinoids investigated consist of a strong ground-state bleach (GSB) peak near the B-band with a broad excited state absorption (ESA) that spans the visible region, on top of which sit a series of smaller Q-band bleach peaks. In the case of OEP **1**, GSB signals from all Q-bands overcome ESA signals and reach negative Δ*A* values. Similar features have also been reported earlier for other free-base porphyrins and oxochlorin as well.^17,39^ Although the relaxation behavior of the ground-state bleach signal is the same, the ESA signal differs for the different molecules. As shown in Figure 3, in the case of oxochlorin **2** and dioxobacteriochlorins **3** and **4**, the GSB signal of Q*_y_*(0, 0) overcomes ESA and shows a prominent GSB feature in accordance with the steady-state absorption spectra. In oxochlorin **2**, the ESA signal decays at different time scales in the wavelength regime between 425 and 500 nm. In the case of dioxobacteriochlorin **4**, the kinetics between 425 and 455 nm show recovery within the initial hundreds of ps before the signal gradually increases and persists within the measured time window. In comparison, the ESA signals between 475 and 550 nm only show gradual decreases within the scan range. As exemplified for the cases of oxochlorin **2** and dioxobacteriochlorin **4**, the transient signals in the fs–ns regime in the range of 425–455 nm show a delayed increase that persists beyond the ns time scales. The delayed increase to the red of the main Soret band, while the ESA signals decrease elsewhere, points to the formation of triplet states. Decay-associated spectral (DAS) analysis shows a similar spectral feature (but less obvious) for dioxobacteriochlorin **3** as well as dioxoisobacteriochlorins **5** and **7** (see Figure S-3), which implies that we are forming triplet states in each of these molecules.

Analyzing our fs–ns TA results, we find that the fitting parameters extracted from probe kinetics can also be used to achieve a satisfactory fit for the Eigen-kinetics calculated from singular value decomposition technique (SVD) analysis (see Table 2 and Figure S-3). In the case of OEP **1**, five exponential decay functions are needed to fit the probe kinetics, whereas, for oxidized OEP samples, four suffice. To fit the tail end of the kinetics from fs–ns TA spectra, a nondecaying component is needed, which will be discussed in the coming section on ns–ms TA. The initial time component of OEP **1** is a rise component on the order of ∼500 fs and contributes majorly to ESA kinetics in 424 nm. But, the amplitude of this component decreases in the redder wavelengths. Similar components with a time scale <1 ps are also present for both the isomers of dioxobacteriochlorins **3** and **4** and dioxoisobacteriochlorin **5**. Baskin et al. reported that the shortest component (100–200 fs lifetime) seen in TA kinetics for H_2_-TPP as the intramolecular vibrational redistribution (IVR) time scales, within which initially deposited energy in a coherent subset of the vibrational phase space spreads to a larger set of states under the influence of intramolecular mode couplings.^39^ It has been also reported in the literature that the IVR time scale varies from a few femtoseconds to 1 ps depending on the nature of the peripheral substitutions of the tetrapyrrole ring.^37,39−41^ Thus, we attribute this component to VR within the Q-bands. The absence of this component in the dioxoisobacteriochlorins **5**–**7** indicates that it occurs on a faster and thus unresolved time scale.

**Table 2:** Fitting Parameters from the Global Fitting of TA Probe Kinetics for OEP **1**, Oxochlorin **2**, Dioxobacteriochlorins (**3** and **4**), and Dioxoisobacteriochlorins (**5**–**7**)^d^

sample	τ_1_^a^ (ps)	τ_2_^a^ (ps)	τ_3_^a^ (ps)	τ_4_^a^ (ps)	τ_5_^a^ (ns)	τ_6_^a^ (μs)	τ_7_^a^
**1**	0.63 ± 0.02^b^	14 ± 1^b^	58 ± 5^b^	316 ± 22^b^	10.6 ± 0.1^b^	n.-d.^b^	n.-d.^c^
					12.4 ± 0.4^c^	146 ± 3^c^	
**2**		15 ± 1^b^	69 ± 18^b^		5.7 ± 0.5^b^	n.-d.^b^	n.-d.^c^
					5.2 ± 0.1^c^	254 ± 8^c^	
**3**	0.19 ± 0.02^b^	14 ± 1^b^			2.8 ± 0.1^b^	n.-d.^b^	n.-d.^c^
					3.1 ± 0.1^c^	163 ± 3^c^	
**4**	0.42 ± 0.01^b^	19 ± 1^b^			2.4 ± 0.1^b^	n.-d.^b^	n.-d.^c^
					2.2 ± 0.1^c^	172 ± 11^c^	
**5**	0.19 ± 0.01^b^	19 ± 1^b^			2.8 ± 0.3^b^	n.-d.^b^	-
					2.4 ± 0.1^c^	22 ± 2^c^	
**7**		11 ±1^b^			1.9 ± 0.1^b^	n.-d.^b^	n.-d.^c^
					1.8 ± 0.1^c^	109 ± 4^c^	
**6**			89 ± 4^b^		3.3 ± 0.2^b^	n.-d.^b^	
					2.4 ± 0.1^c^	97 ± 2^c^	n.-d.^c^

a All measurements were done in a degassed solution.

b Obtained on a setup with ∼150 fs time resolution and a maximum delay of 7 ns.

c Obtained on a setup with ∼1 ns time resolution and a maximum delay of 1 ms.

d n.-d. correspond to a nondecaying component.

A decay component in between 10 and 18 ps is observed for all samples, except for dioxoisobacteriochlorin **6**. DAS spectra (see Figure S-3) calculated from SVD have also suggested that this component can be associated with the shift of the Q*_y_* band for oxochlorin, dioxobacteriochlorins, and dioxoisobacteriochlorins. It has been reported in the literature that the renormalization of the highest occupied molecular orbital–lowest unoccupied molecular orbital (HOMO–LUMO) energy gap due to photoexcitation-induced thermal expansion and subsequent gradual recovery of the porphyrin macrocycle is possible through the vibrational relaxation mechanism via coupling with the solvent.^42^ To verify a similar assignment, we have tracked and fitted the temporal shift of the position of the Q*_y_*(0, 0) GSB peak. A custom-made MATLAB code has been used for this purpose, and the Q*_y_*(0, 0) position has been plotted as a function of delay for OEP **1**, oxochlorin **2**, and dioxobacteriochlorin **4** (see Figure S-4). The GSB peak position of Q*_y_*(0, 0) shows a swift red shift and a subsequent blue shift. The multiexponential fitting of the temporal behavior of the GSB peak position of Q*_y_*(0, 0) has resulted in a ∼13 ps component of significant contribution similar to the 10–18 ps decay component present in probe kinetics. Thus, we assign the ∼10 to 18 ps component in TA to the vibrational cooling (VC) of the porphyrin macrocycle by coupling through the solvent. Single-crystal X-ray diffraction measurements revealed that molecules **1–5** and **7** are mostly planar in nature except for dioxoisobacteriochlorin **6**, which possesses a modest but notable saddling (B_2u_ normal coordinate) distortion.^25^ Thus, the 89 ps component for dioxoisobacteriochlorin **6** may originate from the conformational relaxation of the out-of-plane saddling distortion of the porphyrin macrocycle.^41^ On the other hand, the DAS for OEP **1** and oxochlorin **2** suggests the presence of a rise component of 50–70 ps. During that same time frame, we also monitor a small spectral shift of the Q-bands. Hence, the 50–70 ps rise component may be related to VC. However, in the tetrapyrrole system, it is known that VC usually occurs within a 10–20 ps time scale.^37,39,40^ Nevertheless, Hemant et al. reported a ∼105 ps rise component in the TA spectra of meso-tetrakis pentafluorophenyl porphyrin, which was attributed to either a collective or independent effect of vibrational cooling, conformational relaxation, and intramolecular charge transfer.^43^ On the other hand, time-resolved vibrational spectroscopy was used previously to distinguish between these different processes.^44^

The ns component ideally matches the emission lifetime measured via spectrally resolved TCSPC in each sample. This correspondence confirms the assignment of this ns component to the S_1_ state lifetime. The ISC rate (*k*_ISC_) for each chromophore was computed (see Table 2) using this monitored S_1_ lifetime as well as the internal system crossing quantum yield (ϕ_ISC_, described in Table 1).^35^ We observed a systematic 2-fold increase in the ISC rate from OEP **1** with each addition of a β-oxo substituent, thus reaching a 4-fold increase in ISC with the doubly oxygenated porphyrinoids relative to the ISC rate observed for OEP.

#### 3.2.2 Transient Absorption Spectroscopy from ns to ms

Transient absorption spectroscopy in the ns–ms time scale was performed in anaerobic conditions. The differential absorbance at several delay points along with reconstructed kinetics from fs to ms for OEP **1**, oxochlorin **2**, dioxobacteriochlorins **3** and **4**, and dioxoisobacteriochlorins **5**–**7** is plotted in Figure 3. Transient spectra are marked by the GSB peaks of Q-bands on the top of broad excited-state absorption. Spectral features at an early time represent singlet spectra, which evolve into triplet spectra at subsequent times via ISC. The decay components from the global fitting of probe kinetics with multiexponential function are shown in Table 2 (Figure S-3). We have assigned the resolved μs lifetime components to the triplet (T_1_) lifetimes. A similar time scale of triplet lifetime has been reported in the case of other oxochlorin, bacteriochlorin, and porphyrinoid molecules.^13,19^ This lifetime increases from 146 ± 3 μs for OEP **1** to 254 ± 8 μs for oxochlorin **2** and increases to 163 ± 3 and 172 ± 11 μs for dioxobacteriochlorins **3** and **4**, respectively. On the other hand, this component decreases to 108 ± 4, 97 ± 2, and 22 ± 1 μs for dioxoisobacteriochlorins **5**–**7**, respectively, as shown in Figure 3c. We note that for all of the samples, except for dioxoisobacteriochlorin **5**, we monitored a transient signal at negative time delays. This background signal, which was subtracted during postprocessing, is indicative of a long-lived (>ms) component, which is denoted as nondecaying in Table 2. In the case of the isomers of dioxoisobacteriochlorins **5–7**, the amplitude of this nondecaying component is 2 orders of magnitude less than that of the earlier components and is thus negligible. However, in the case of OEP **1**, oxochlorin **2**, and bacteriochlorins **3–4**, this component is about the same order of magnitude as that of the latest resolved components.

##### Interplay between the Spin–Vibronic Channel and NH Tautomerism

**Figure 4:**
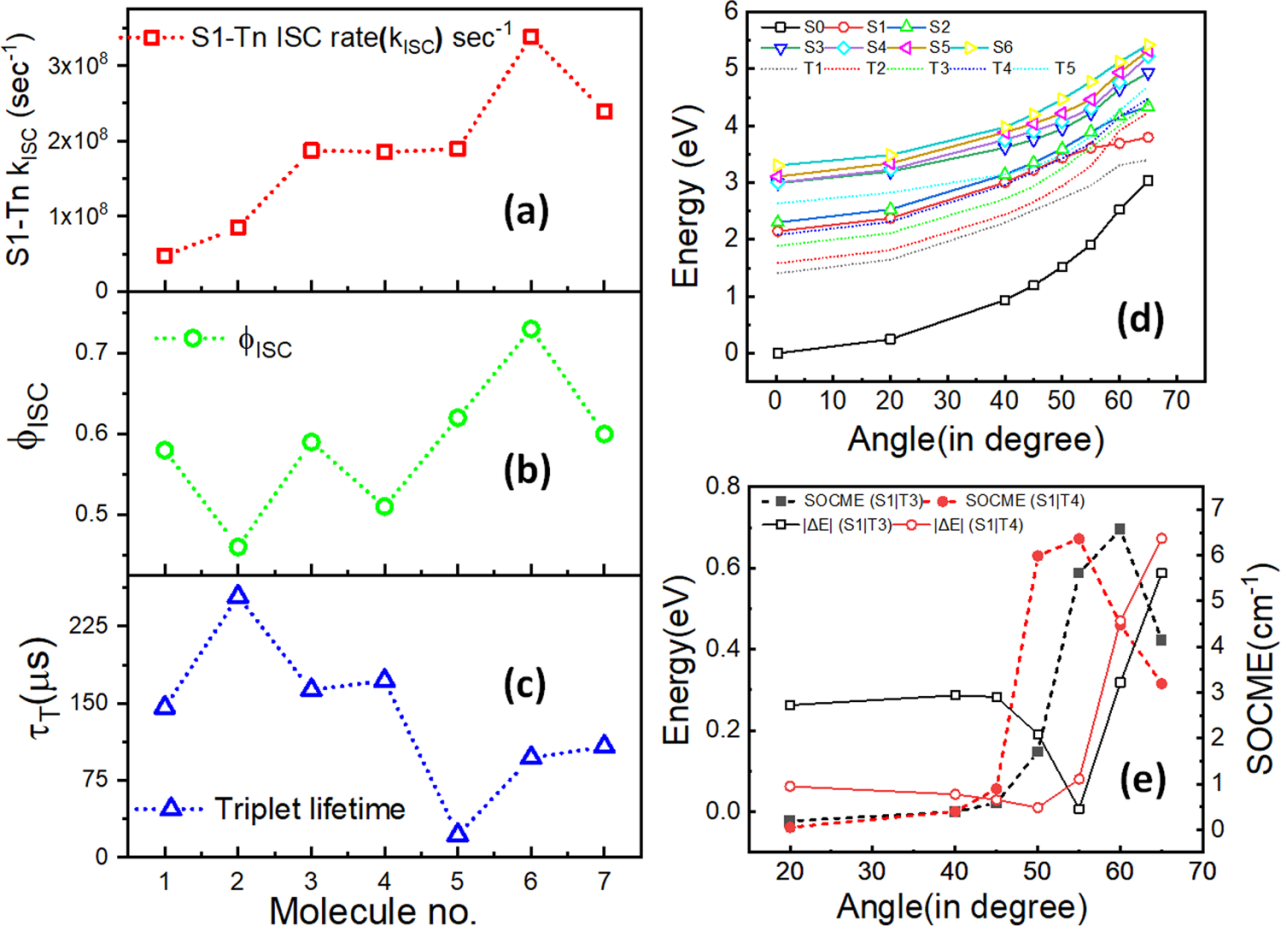
(a) ISC rate (*k*_ISC_), (b) ISC quantum yield (ϕ_f_), and (c) triplet lifetime (τ_T_) for OEP **1**, oxochlorin **2**, dioxobacteriochlorins **3** and **4**, and dioxoisobacteriochlorins **5–7**. (d) Potential energy curves for singlet (solid) and triplet states (dashed) along the C=O out-of-plane displacement for oxochlorin **2**. (e) Energy differences and spin–orbit couplings along the out-of-plane displacements of the carbonyl group between S_1_ and T_3_ and S_1_ and T_4_ state pairs calculated at the B3LYP/def2-SV(P) level. Note that the numbering of the states increases with their energy. Hence, S_0_ corresponds to the ground state, and S_1_ and S_2_ correspond to the first and second lowest singlet excited state (i.e., Q-bands), respectively.

**Figure 5:**
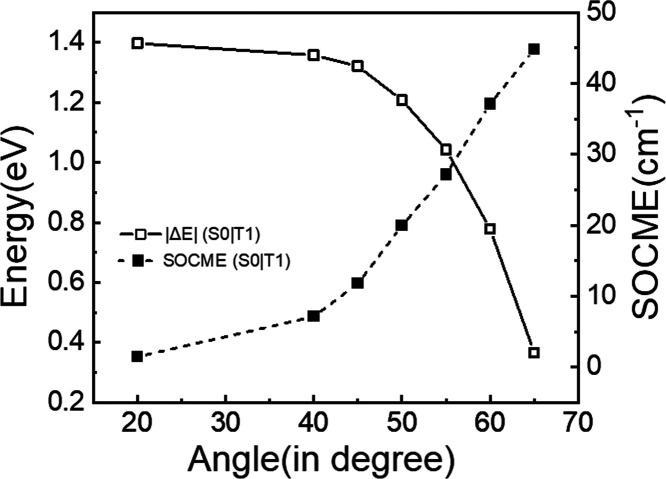
Energy differences and spin–orbit couplings along the out-of-plane displacements of the carbonyl group S_0_–T_1_ state pair in oxochlorin **2** calculated at the B3LYP/def2-SV(P) level.

As evidenced in the previous sections, we have experimentally resolved a clear trend of increasing ISC rate with an increasing number of carbonyl β-oxo substituents. In free-base porphyrins, the ISC rate is determined by the energy gap between the states involved and the strength of the spin–orbit coupling, which can be quantified by calculating the spin–orbit coupling matrix element (SOCME).^10^ On the other hand, the calculated SOCME of the lowest single and triplet states from several porphyrin derivatives are found to be extremely small.^10^ Furthermore, theoretical calculations have predicted that the out-of-plane normal modes in free-base porphyrin macrocycles play an essential part in these relaxation dynamics by enhancing the SOCME.^21,32^ To understand how the spin–vibronic channel affects the ISC rate in the β-oxo-chromophores, DFT calculations were performed to estimate the potential energy curve for the singlet and the triplet state as a function of the angle between the carbonyl group and the molecular plane. Calculations were performed on oxochlorin **2** (Figures 4 and 5) as well as on dioxobacteriochlorin **4** (Figures S-5 and S-7) and dioxoisobacteriochlorin **6** (see Figures S-6 and S-8). Molecules **2**, **4**, and **6** are taken as representative for the oxochlorin, bacteriochlorin, and isobacteriochlorin categories, respectively.

All of the calculations have shown that the energy difference between the potential energy (PE) landscape of the S_1_ state and higher energy triplet states decreases when the carbonyl angle increases. Furthermore, calculations show crossings of PE between the S_1_ and higher triplet states when the out-of-plane carbonyl distortion angle reaches ∼45°, as shown in Figure 4d for oxochlorin **2**.

Equivalent crossings were also observed for the isomers of dioxobacteriochlorins and dioxoisobacteriochlorin considered (see Figures S-6 and S-7). The calculated SOCME along the out-of-plane carbonyl distortion shows that the maximum coupling between the S_1_ state and the T_3_ and T_4_ states occurs in the range of 50–60°, which coincides with the energy gap minima between these same states (Figures 4e, S-6, and S-7). Accordingly, the maximum SOCME and minimum energy gaps correlate well with the increased ISC rates monitored for the oxidized derivatives of OEP. It is interesting to note that the crossings occur between S_1_ and higher energy triplet states and never directly with the low-lying triplet T_1_, as previously reported for chlorins and bacteriochlorins.^45^ The relatively large calculated spin-orbit couplings for these species are in line with the recently described effect of spin-orbit coupling amplification in hydrogenated porphyrin rings due to an increase of the superposition of the hole and electron densities between the S_1_ and T_n_ states in these molecules.^45^ Thus, the systematic 2-fold increase in the ISC rate from porphyrin **1** to oxochlorin **2** and a further 2-fold increase to the doubly oxygenated porphyrinoids are interpreted as an increased value of the SOCME between S_1_ and triplet states with the out-of-plane carbonyl distortion. We do not deny the possibility that other affected modes also play a role in controlling the ISC rate, but the C=O out-of-plane distortion mode is expected to be the most influential.

In addition to affecting ISC rates, the presence of carbonyl β-oxo substituents is also expected to affect triplet lifetimes. Our spin–vibronic DFT calculations indeed suggest that oxochlorin and dioxochlorin display a decrease in the energy difference between the S_0_ and T_1_ states as the angle between the carbonyl group and the molecular plane increases (Figures 4d, S-6, and S-7). More specifically, as shown in the representative case of oxochlorin **2**, the decrease in the S_0_ – T_1_ energy difference induced by the carbonyl vibration is also accompanied by an increase in spin–orbit coupling between these states (see Figures 5, S-8b, and S-9b). This pattern points toward the presence of crossing points between S_0_ and T_1_ for vibrational angles above 65°. And, in turn, the presence of crossing points is expected to result in shorter T_1_ lifetimes. Shorter T_1_ lifetimes, however, have not been experimentally observed for oxochlorin **2** and dioxobacteriochlorins **3** and **4**. The fact that these molecules behave contrarily to our expectations either indicates that the carbonyl out-of-plane vibration is not the dominant factor that affects T_1_ lifetimes or simply that the expected crossing points are never reached. We do, however, monitor triplet lifetimes that are significantly shorter for dioxobacteriochlorins **5**–**7**, compared to those for OEP **1**. Triplet lifetimes for bacteriochlorins **5**–**7** were found to be 22 ± 2, 108 ± 4, and 97 ± 2 μs, respectively, in comparison to 146 ± 3 μs for OEP **1**. Interestingly, these are the only molecules in our series that are found in different tautomeric forms, as revealed by molecular circular dichroism (MCD) and TDDFT calculations.^25^ The presence of ground-state tautomers implies the presence of excited-state tautomers as well, and it has been previously shown that excited-state single and double proton transfer do affect relaxation dynamics.^32,46^ It was shown that for dioxoisobacteriochlorin **5**, there exist three tautomeric forms labeled **5ac**, **5ad**, and **5bd**, with a–d corresponding to the proton positions, as shown in Figure 1, with a ratio of 9:72:19%, respectively. Dioxoisobacteriochlorin **6** is found in two tautomeric forms **6ab:6ac** with proportions 96:4% and dioxoisobacteriochlorin **7** is found in two tautomeric forms **7ac:7ad** with proportions 73:27%.^25^ To better understand the unexpected trend in triplet lifetimes, we have also investigated C=O stretching (see Figure S-11) and intra-ring carbon–carbon stretching modes (see Figure S-12) for possible T_1_–S_0_ crossings. However, the energy gap between these states remains very large (∼1.3 eV) throughout the vibration coordinates. According to these modeling, the reduction in triplet lifetimes monitored for dioxobacteriochlorins **5–7** is thus attributed to the presence of ground-state tautomers rather than to the number of carbonyl β-oxo substituents. We cannot eliminate the potential role played by the position of the β-oxo substituents in tuning the triplet lifetimes, but the T_1_–S_0_ PE calculations are expected to give similar results regardless of the β-oxo substituent position. The presence of NH tautomerism, because it seems to play a pre-eminent role in tuning the triplet lifetime, represents a constraint that is not under our control. However, this complication will become moot in metallated β-oxo-porphyrins. Accordingly, the role of the position of β-oxo substitutions will be further investigated in metallated β-oxo-porphyrins in a forthcoming study.

## 4 Conclusions

In conclusion, we demonstrated that the modification of octaethylporphyrin with one or two β-oxo-substituents provides an effective way to tune its photophysical properties. More specifically, carbonylation results in a red-shifted and increased Q-band absorption. And more importantly, carbonylation results in a systematic tuning of the ISC rates, with a 2-fold increase for every additional oxo-substituent, irrespective of their position around the macrocycle. TDDFT modeling shows that this systematic increase of the ISC rate can be directly related to an increased number of S_1_–T_n_ crossings induced by the spin–vibronic channels of the carbonyl out-of-plane distortion modes. Additionally, we have shown that triplet lifetimes are also affected. But, for such longer-lived states, it is the presence of tautomers in dioxoisobacteriochlorins that seems to be the main driver, irrespective of the number of oxo-substitutions. The possible effect of the positions of the oxo-substitutions onto the triplet lifetime will be the focus of a forthcoming study. For the time being, β-oxo-substitutions have been shown to provide a nontoxic and economical way to systematically tune the steady-state features and ISC rates.

## Supporting Information Available

Vertical excitation energies in nm and oscillator strengths for different porphyrins; Q-band transitions and its description in terms of molecular orbital excitations percentage contributions; B band transitions and its description in terms of molecular orbital excitation percentage contribution; TCSPC data; SVD of fs-TA data; potential energy curves for singlet and triplets along the C–O out-of-plane displacement for dioxobacteriochlorin and dioxoisobacteriochlorins; and potential energy curve for oxochlorin along the C–O stretch (PDF)
