# Supplementary material for: Tailoring the Intersystem Crossing and Triplet Dynamics of Free-Base Octaalkyl-β-oxo-Substituted Porphyrins: Competing Effects of Spin–Vibronic and NH Tautomerism Relaxation Channels
Source: J Phys Chem A. 2022 Mar 29;126(16):2522–31. doi: 10.1021/acs.jpca.2c01225 (PMC9059185; doi:10.1021/acs.jpca.2c01225)
Supplement: Supplementary file 1 — jp2c01225_si_001.pdf [file jp2c01225_si_001.pdf]

Supporting Information

Tailoring the Intersystem Crossing and Triplet Dynamics of Free Base Octaalkyl-β-oxo-substituted Porphyrins: Competing Effects of Spin-Vibronic and NH Tautomerism Relaxation Channels

Sayantan Bhattacharya[*†1], Arthur Graf[†1], Anna Karolyna M. S. Gomes[2], Nivedita Chaudhri[3], Dimitri Chekulaev[1], Christian Brückner[1], Thiago M. Cardozo[2] and Adrien A. P. Chauvet[*1]

1 Department of Chemistry, The University of Sheffield

2 Instituto de Química (IQ), Universidade Federal do Rio de Janeiro

3 Department of Chemistry, University of Connecticut

* Corresponding authors

† Contributed equally

Table S-1 - Vertical excitation energies in nm and oscillator strengths (in parenthesis) for different porphyrins obtained at the B3LYP/def2-SVPD level

	H_2 OEP	Oxochlorin 2	Bacteriochlorin 4	Isobacterio-chlorin 6
S_1	558.3 (0.04)	570.6 (0.05)	592.4 (0.11)	561.2 (0.02)
S_2	525.3 (0.09)	534.0 (0.01)	541.4 (0.01)	556.5 (0.06)
S_3	406.2 (0.30)	412.1 (0.13)	411.0 (0.02)	458.6 (0.00)
S_4	399.5 (0.01)	411.5 (0.01)	409.7 (0.02)	407.1 (0.01)
S_5	383.5 (0.28)	395.7 (0.31)	394.8 (0.62)	394.0 (0.08)
S_6	380.7 (0.00)	372.1 (1.16)	386.8 (0.92)	389.2 (0.81)
S_7	358.4 (1.01)	365.1 (0.19)	381.0 (0.03)	381.1 (1.05)
S_8	348.8 (1.17)	364.1 (0.01)	380.8 (0.11)	349.4 (0.01)
S_9	322.3 (0.01)	338.6 (0.01)	350.9 (0.00)	338.7 (0.20)
S_{10}	319.5 (0.00)	334.3 (0.03)	343.6 (0.01)	322.4 (0.01)

Note that in **Table S-1**, the two states with higher oscillator strengths are the main contributions to the B bands while the states above these correspond to the Q bands.

Table S-2 – Q-bands transitions and its description in terms of molecular orbital excitations percentage contributions obtained at the B3LYP/def2-SVPD level.

Molecule	Transition	Excitation Energy (eV)	Oscillator Strength	Contributions (%)	
**H_2 OEP**	$S_0 \rightarrow S_1$	2.22	0.01	HOMO-1 → LUMO+1	51.6
				HOMO → LUMO	47.3
	$S_0 \rightarrow S_2$	2.36	0.01	HOMO → LUMO+1	53.2
				HOMO-1 → LUMO	45.7
**oxochlorin 2**	$S_0 \rightarrow S_1$	2.17	0.06	HOMO → LUMO	60.6
				HOMO-1 → LUMO+1	29.2
				HOMO-1→LUMO	5.50
	$S_0 \rightarrow S_2$	2.32	0.01	HOMO-1 → LUMO	46.9
				HOMO → LUMO+1	42.8
				HOMO → LUMO	8.10
**bacteriochlorin** 4	$S_0 \rightarrow S_1$	2.09	0.11	HOMO → LUMO	76.0
				HOMO-1 → LUMO+1	23.0
	$S_0 \rightarrow S_2$	2.29	0.01	HOMO→ LUMO+1	53.2
				HOMO-1 → LUMO	46.2
**isobacteriochlorin** 6	$S_0 \rightarrow S_1$	2.21	0.02	HOMO→ LUMO+1	53.1
				HOMO-1 → LUMO	44.7
	$S_0 \rightarrow S_2$	2.23	0.06	HOMO → LUMO	74.0
				HOMO-1 → LUMO+1	23.9

Table S-3 – B-bands transitions and its description in terms of molecular orbital excitation percentage contributions obtained at the B3LYP/def2-SVPD.

Molecule	Transition	Excitation Energy (eV)	Oscillator Strength	Contributions (%)	
H_2 OEP	$S_0 \rightarrow S_7$	3.46	1.06	HOMO-3 → LUMO	40.2
				HOMO → LUMO+1	27.9
				HOMO-1 → LUMO	27.3
	$S_0 \rightarrow S_8$	3.55	1.17	HOMO-3 → LUMO+1	46.0
				HOMO-1 → LUMO+1	26.2
				HOMO →LUMO	24.6
Oxochlorin 2	$S_0 \rightarrow S_5$	3.13	0.31	HOMO-1 → LUMO+1	32.2
				HOMO-2 → LUMO+1	28.6
				HOMO-2 → LUMO	22.2
	$S_0 \rightarrow S_6$	3.33	1.16	HOMO → LUMO+1	42.5
				HOMO-1 → LUMO	37.1
				HOMO-2 → LUMO	11.8
bacteriochlorin 4	$S_0 \rightarrow S_5$	3.14	0.62	HOMO-1 → LUMO+1	60.4
				HOMO → LUMO	17.2
				HOMO → LUMO+2	10.1
	$S_0 \rightarrow S_6$	3.21	0.92	HOMO-1 → LUMO	41.6
				HOMO → LUMO+1	37.2
				HOMO → LUMO+3	4.40
bacteriochlorin 6	$S_0 \rightarrow S_6$	3.19	0.81	HOMO-1 → LUMO+1	69.3
				HOMO →LUMO	21.9
	$S_0 \rightarrow S_7$	3.25	1.05	HOMO-1 → LUMO	44.5
				HOMO → LUMO+1	37.3
				HOMO-2 → LUMO+1	8.4

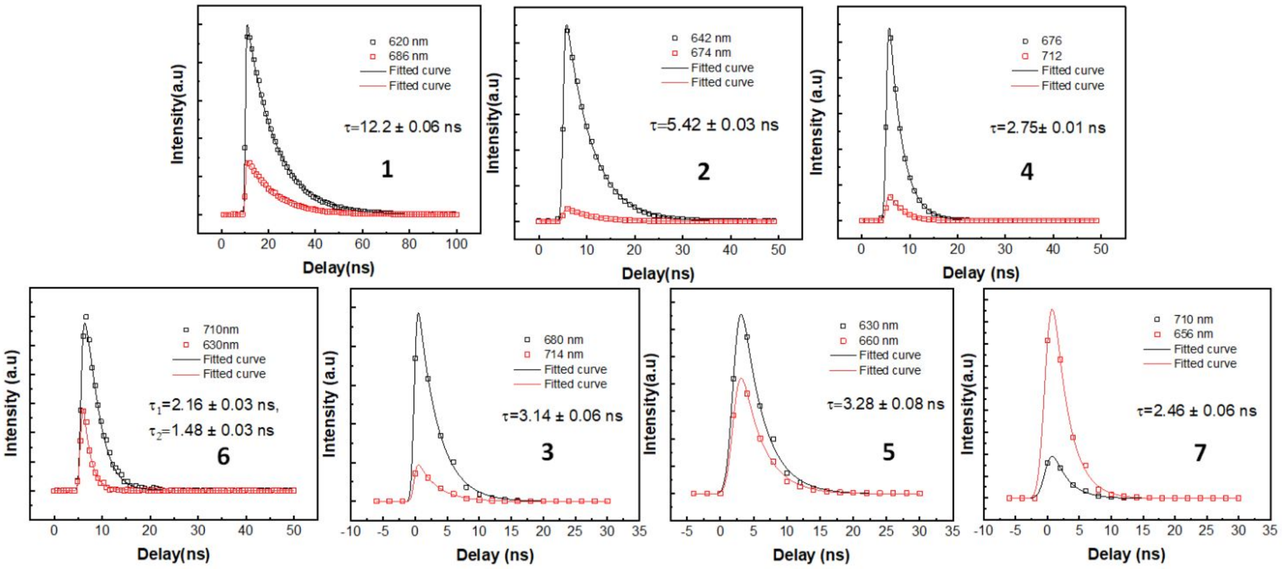

Figure S-1 Global fitting of emission kinetics of H_2-OEP **1**, oxochlorin **2,** bacteriochlorins (**3,4**) and isobacteriochlorins (**5,6,7**) measured using spectrally resolved TCSPC

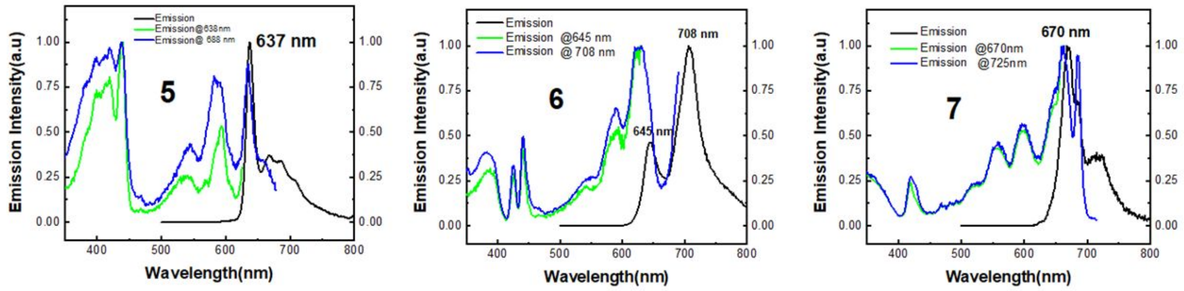

Figure S-2 Excitation spectra along with emission spectra for all the isomers of dioxoisobacteriochlorin **5, 6** and **7.**

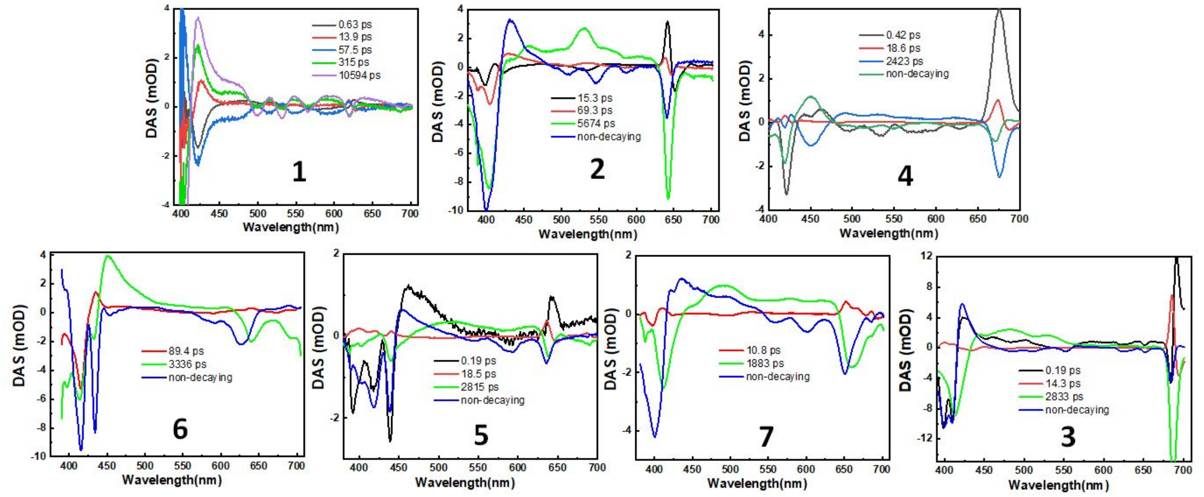

Figure S-3 Decay associated spectra (DAS) from SVD H_2-OEP **1**, oxochlorin **2,** bacteriochlorins (**3,4**) and isobacteriochlorins (**5,6,7**)

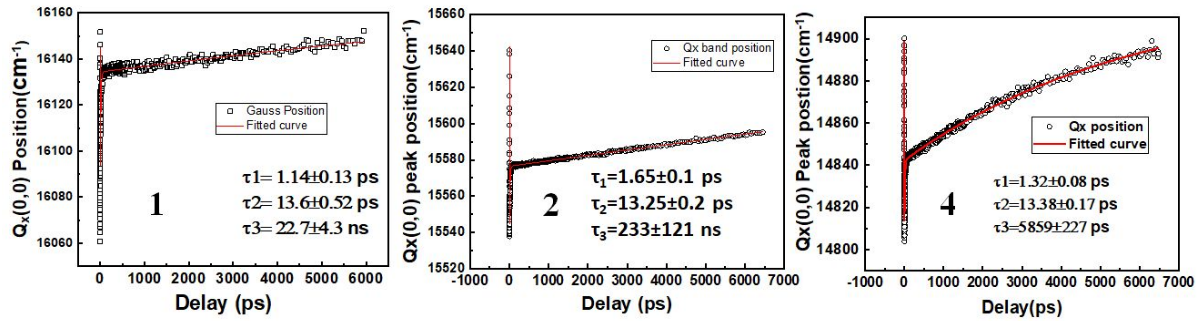

Figure S-4 $Q_y(0,0)$ GSB position has been determined by fitting TA spectra with an inverted gaussian function for consecutive delays. Multi-expoenential fitting of $Q_y(0,0)$ GSB peak position as function of delay for H_2-OEP **1**, oxochlorin **2**, dioxobacteriochlorin **4** has been performed.

For all the following figures, note that the numbering of the states increases with energy. Hence, S_0 corresponds to the ground state, S_1-S_4 correspond to Q-bands, etc.

Figure S-5 Potential energy curves for singlet (solid) and triplets (dashed) along the C-O out-

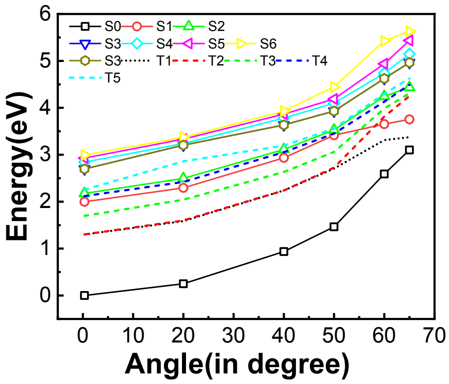

of-plane displacement calculated at the B3LYP/def2-SV(P) level for dioxobacteriochlorin **4**. Note that the numbering of the states increases with energy. Hence, S_0 corresponds to the ground state, S_1-S_4 correspond to Q-bands, etc.

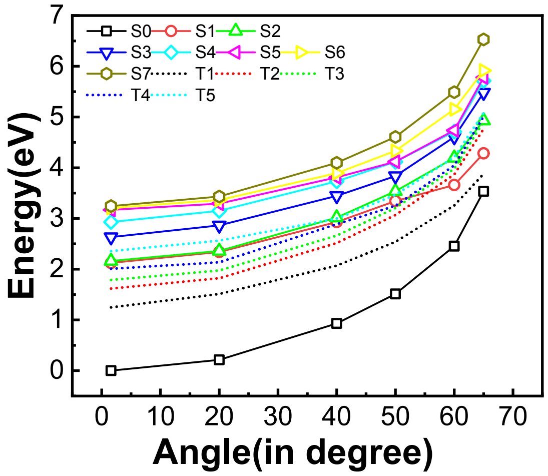

Figure S-6 Potential energy curves for singlet (solid) and triplets (dashed) along the C-O out-of-plane displacement calculated at the B3LYP/def2-SV(P) level for dioxoisobacteriochlorin **6**.

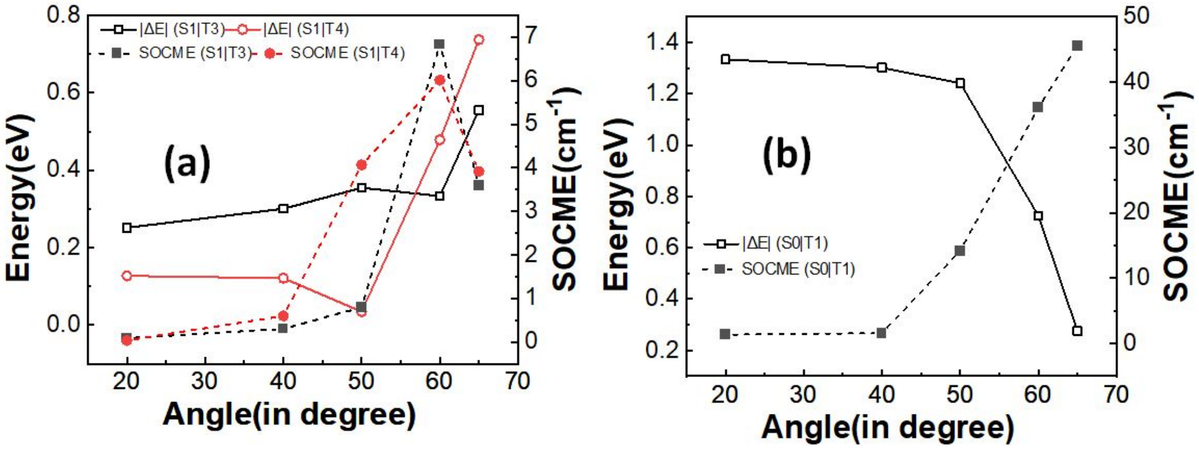

Figure S-7 Energy difference and spin-orbit coupling along out-of-plane displacement of the carbonyl group between a) S_1-T_3 and S_1-T_4 state pairs and b) S_0-T_1 state pair in dioxobacteriochlorin **4**, calculated at the B3LYP/def2-SV(P) level.

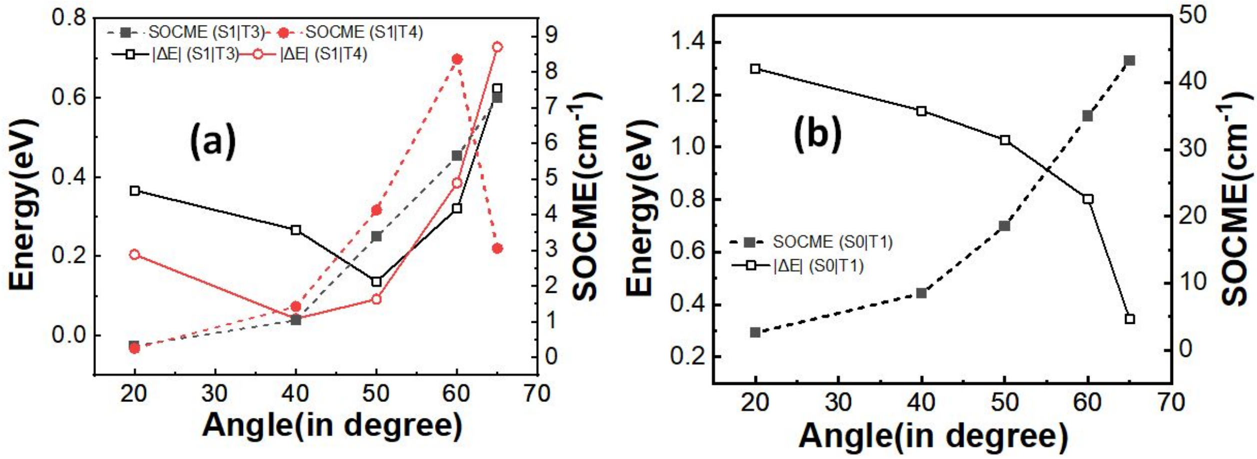

Figure S-8 Energy difference and spin-orbit coupling along out-of-plane displacement of the carbonyl group between a) S_1-T_3 and S_1-T_4 state pairs and b) S_0-T_1 state pair in dioxoisobacteriochlorin **6**, calculated at the B3LYP/def2-SV(P) level.

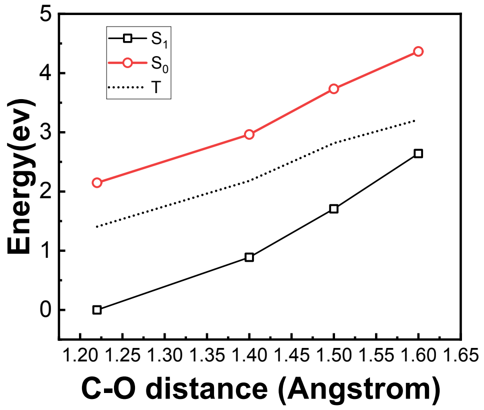

Figure S-9 Potential energy curve for **2**-oxochlorin along the C-O stretch obtained at B3LYP/def2-SVPD level starting from the optimized S_1 state geometry.

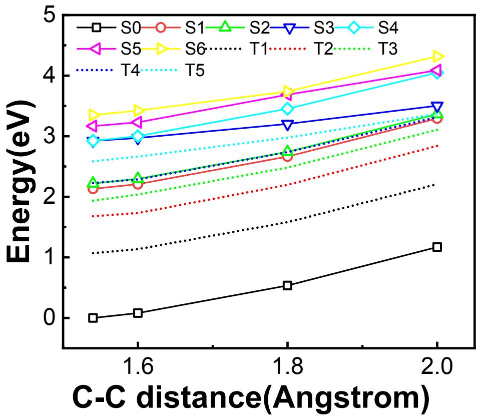

Figure S-10 Potential energy curve along the C-C bond connected to the carbonyl group and the corresponding for **2**-oxochlorin obtained at B3LYP/def2-SVPD level. Dark and light blue lines correspond to the B states while red and pink lines correspond to Q states. Dashed lines indicate triplet states.

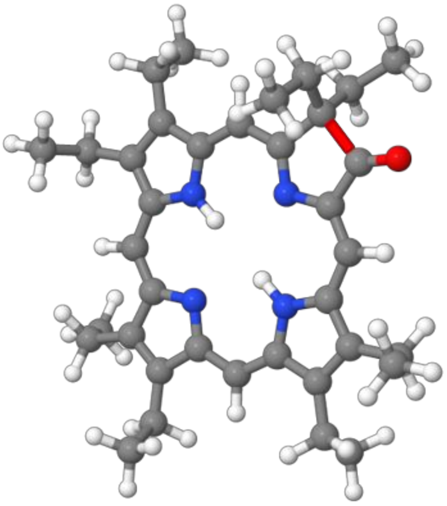

Figure S-11 C-C bond used to construct the C-C stretch PES for **2**-oxochlorin.
